# Modic Changes and Disc Degeneration Caused by Inoculation of* Propionibacterium acnes* inside Intervertebral Discs of Rabbits: A Pilot Study

**DOI:** 10.1155/2016/9612437

**Published:** 2016-01-26

**Authors:** Zhe Chen, Yuehuan Zheng, Ye Yuan, Yucheng Jiao, Jiaqi Xiao, Zezhu Zhou, Peng Cao

**Affiliations:** ^1^Department of Orthopedics, Ruijin Hospital, Shanghai Jiao Tong University School of Medicine, Shanghai 200025, China; ^2^Shanghai Key Laboratory for Prevention and Treatment of Bone and Joint Diseases with Integrated Chinese-Western Medicine, Shanghai Institute of Traumatology and Orthopedics, Ruijin Hospital, Shanghai Jiao Tong University School of Medicine, Shanghai 200025, China; ^3^Department of Orthopedics, Ruijin Hospital North, Shanghai Jiao Tong University School of Medicine, Shanghai 201821, China; ^4^Department of Medical Microbiology and Parasitology, Shanghai Jiao Tong University School of Medicine, Shanghai 200025, China; ^5^Department of Orthopedic Surgery, Xinhua Hospital, Shanghai Jiao Tong University School of Medicine, Shanghai 200092, China

## Abstract

*Purpose*. To investigate whether* P. acnes* could induce disc degeneration and Modic changes when inoculated into the discs of rabbits.* Method*. A wild-type strain of* P. acnes* isolated from a patient associated with Modic change and disc degeneration was inoculated into the intervertebral discs of rabbits. Meanwhile,* S. aureus* was injected into the discs to establish a model of discitis as the comparison and a standard strain of* P. acnes* was inoculated as the control. MRI and histological change were observed.* Results*. Both the* P. acnes-*inoculated and* S. aureus*-inoculated rabbits showed hyperintense signals at endplates and hypointense signals at nucleus pulposus on T2WI. However,* P. acnes* only resulted in moderate disc degeneration and endplates rupture in histological examination, which was different from the pathological change of discitis caused by* S. aureus*. In addition, higher death rates (2/3* versus* 0/5) were observed in* S. aureus*-inoculated rabbits.* Conclusion*. Compared to* S. aureus*, the pathological change caused by* P. acnes* would be considered as Modic-I change and disc degeneration rather than a discitis.

## 1. Introduction

Modic changes are the successive event of disc degeneration [1], presenting as signal intensity changes at cartilage endplates and subchondral bone in MRI [2, 3]. Many studies have claimed a strong connection between Modic changes and nonspecific low back pain and that is why so much attention was given to this pathological change [4, 5]. Unfortunately, the exact pathogenic mechanism of Modic changes is still unclear until now.

One of the explanations was the bacterial mechanism. It was reported that some low-virulent anaerobic bacteria could be isolated from the intervertebral discs only with degeneration but without pyogenic change [6, 7]. Subsequently, the low-virulent anaerobic bacteria were suggested as the pathogens for Modic changes and low back pain [8, 9]. A randomized controlled trial showing that patients acquired excellent pain relief and Modic-I changes attenuation after oral antibiotics treatment further validated this theory [10].

Among those low-virulent anaerobic bacteria,* Propionibacterium acnes* (*P. acnes*) was the major and most frequent isolated bacterium, accounting for approximately 85%, so that it ought to be the most possible pathogen for Modic change and low back pain [6, 8]. It is an anaerobic-aerotolerant Gram-positive rod-shaped bacterium, residing in the skin, oral cavity, and conjunctiva as part of the normal flora [11]. More importantly, it was also an opportunistic pathogen responsible for series of bone and joint infection [11].

Nevertheless, there has been controversies and argumentation against the relationship between bacteria and Modic changes, highlighting that the isolated* P. acnes* was the contamination during tissue harvest rather than the original colonization inside intervertebral discs [12]. In addition, the lack of deterministic causal evidence based on animal studies was another major defect [13]. To solve this problem, Koch's Postulates, which demonstrate that pathogenic microbes isolated from patients could cause the same disease in animal models, were a reasonable method [14, 15].

Therefore, the primary goal of this investigation was to test whether the wild-type strain of* P. acnes* isolated from the intervertebral disc of patient associated with Modic changes and disc degeneration has the ability to induce the same pathological change when inoculated into the discs of rabbits. In the meantime, a pyogenic discitis model caused by* Staphylococcus aureus* (*S. aureus*) was established to make a comparison and a standard strain of* P. acnes* was used as the control. To our knowledge, this is the first animal experiment to demonstrate the possible causative relationship between* P. acnes* and Modic changes, which is very important to reveal the exact role low-virulence anaerobic bacteria are playing in intervertebral discs.

## 2. Materials and Methods

### 2.1. Animals

The harvest of intervertebral disc from human being was approved by the clinical Ethics Committee of Ruijin Hospital, Shanghai Jiao Tong University School of Medicine. Animal experiments were approved by the Animal Ethics Committee of Shanghai Jiao Tong University School of Medicine. A total of eight New Zealand rabbits weighing around 2.0~2.5 kg each were included in this study (Table 1). They were fed with regular water and food and kept for at least one week before the surgery for acclimatization.

### 2.2. Isolation and Identification of the Wild-Type Strain of* P. acnes*

**Figure 1:**
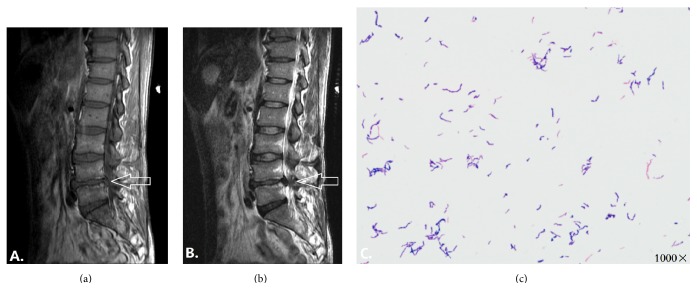
The wild-type strain of* P. acnes* was isolated from the patient associated with severe disc degeneration and Modic change at the segment of L4~L5. (a)~(b) MRI examination of the patient showed signal intensity changes on the segment of L4~L5 at the cartilage endplate on T1WI and T2WI (a, b), suggesting a Modic change (b). Both were indicated by a white arrow. Meanwhile, severe disc degeneration and disc herniation were found at the same segment. (c) Gram's staining depicted a Gram-positive rod-shaped bacterium after three-day culture in anaerobic blood plate.

A wild-type strain of* P. acnes* was isolated from a patient who initially took part in the epidemiological study about the relationship between* P. acnes* and disc degeneration [16]. The patient, a 48-year-old Chinese male, underwent discectomy due to sciatica and low back pain and did not show any symptoms of discitis (no fever, no body weight loss, and no abnormal ESR). MRI revealed a severe disc herniation and Modic change at intervertebral discs of L4~5 (Figures 1(a) and 1(b)).

For the process of isolation of the bacterium, disc material of L4~L5 was harvested under stringent sterilized situation and some muscle and ligaments around the disc were also obtained as contamination marker. All samples (disc material and control samples) were enriched in a 9 mL sterilized Tryptone Soya Broth (TSB, BD, NJ, USA) mixed with 1 mL of bovine serum (Gibco, Life Technologies, CA, USA). The broth was cultured inside an anaerobic Glove Box device (Yuejin Medical Instruments Corporation, Shanghai, China) for fourteen days with circumstance of 37°C, 80% N_2_-10% CO_2_-10% H_2_ mixed gas. When limpid broth turned into cloudy, it was transferred into the Anaerobic Blood Agar (Beiruite Bio-technology, Zhengzhou, China) for another three-day culture under the same anaerobic circumstance.

Following previous method [8], the purified single bacterial colony was identified using Gram's staining, analytical profile index (API) biochemical analysis of Rapid ID 32A (bioMérieux, France), and polymerase chain reaction (PCR) amplification of 16SrDNA. The procedures of 16SrDNA-PCR followed previous method [8, 16]. Finally, a wild-type strain of* P. acnes* was isolated from the intervertebral disc of the patient and no bacterium was found at the muscle and ligament, suggesting that the isolated* P. acnes* originated from the intervertebral discs rather than from the contamination of the incision. Moreover, this microbe was confirmed as* P. acnes* by these three identification methods (Figure 1(c)).

### 2.3. Preparation of Bacterial Inoculum

The wild-type strain of* P. acnes* harvested from a single colony on the agar was washed for three times using TSB without bovine serum and then suspended in the same solvent (sterilized TSB without bovine serum) with adjusting the concentration to 1 × 10^7^ CFU (Colony-Forming Units) per mL using the plate count method. In the meantime, a standard strain of* P. acnes* was obtained from the Guangdong Microbiology Culture Center (ATCC: 6919, GIM: 1.243, Guangdong, China) and prepared into the same suspension as the method described above. Additionally, a single colony of* S. aureus* (ATCC 25923, granted by Department of Medical Microbiology and Parasitology, Shanghai Jiao Tong University School of Medicine) harvested from Columbia blood agar plate was suspended in sterilized saline with the same concentration of 1 × 10^7^ CFU per mL.

### 2.4. Surgical Procedure

All rabbits were anesthetized using 2.5% sodium pentobarbital with 1 mL/kg via auricular vein and were then placed in right lateral position. The skin was shaved and sterilized carefully with povidone iodine for three times. Prior to the surgery, the desired vertebrae (L5, L6, and L7) and intervertebral discs (L5~L6 and L6~L7) were identified using CT. Meanwhile, the diameters of the desired intervertebral discs on coronal plane were measured using CT to determine the depth of penetration.

A posterolateral retroperitoneal approach was conducted to expose the desired vertebrae (L5, L6, and L7) and discs (L5~L6 and L6~L7) [17]. Before inoculation, plenty of sterilized saline was used to wash the incision to prevent contamination. A microsyringe with 28-gauge needle (Hamilton, Nevada, USA) was used for inoculation and the penetration depth was fixed at 5~6 mm based on the measured parameters. A volume of 25 *μ*L wild-type strain or standard strain of* P. acnes* suspension was inoculated into the nucleus pulposus at the segment of L6~L7 and 25 *μ*L of sterilized TSB without bovine serum was injected at the segment of L5~L6 as internal control at the same time (Table 1). The holes of penetration were sealed with bone wax to prevent the leakage of inocula and the fascia and skin were closed layer by layer with silk sutures. Collectively, three animals received the wild-type strain of* P. acnes* isolated from the patient and the other two animals were inoculated with the standard strain of* P. acnes*. With the same method, the other three rabbits received the inoculation of 25 *μ*L* S. aureus* suspension at the segment of L6~L7 (Table 1). All animals were fed regularly and no antibiotics were used before and after the surgery.

### 2.5. MRI Examination

MRI was performed before and every two weeks after the inoculation until the end of follow-up at the eighth week. Briefly, animals were fasted closely on a wooden plate at prone position after deep anaesthetization and scanned with knee coil in a 3.0-T GE HDxt Signa/MRI system (GE Corporation, Connecticut, USA). The parameters were as follows: T1W TE/TR, 8.4 ms/540 ms, T2W TE/TR, 48.2 ms/2000 ms, slice thickness, 3 mm, and Field of View, 20 × 20. All MRI images were evaluated by two authors independently (Z. Chen and Z. Zhou). According to Pfirrmann classification system, the degree of intervertebral discs degeneration was evaluated and classed into five grades [18].

### 2.6. Histological Examination

Animals were euthanized at the eighth week for histological examination. Briefly, intervertebral discs with endplates were fixed in 4% formaldehyde for 24 hours and then decalcified with EDTA (Ethylenediaminetetraacetic Acid) for three months, processed with routine paraffin embedding, and sectioned at 5 *μ*m. Hematoxylin and eosin staining was conducted and digital pictures were captured under the magnification of 100x.

## 3. Results

### 3.1. Signal Changes in MRI at* P. acnes*-Inoculated and* S. aureus*-Inoculated Intervertebral Discs

**Figure 2:**
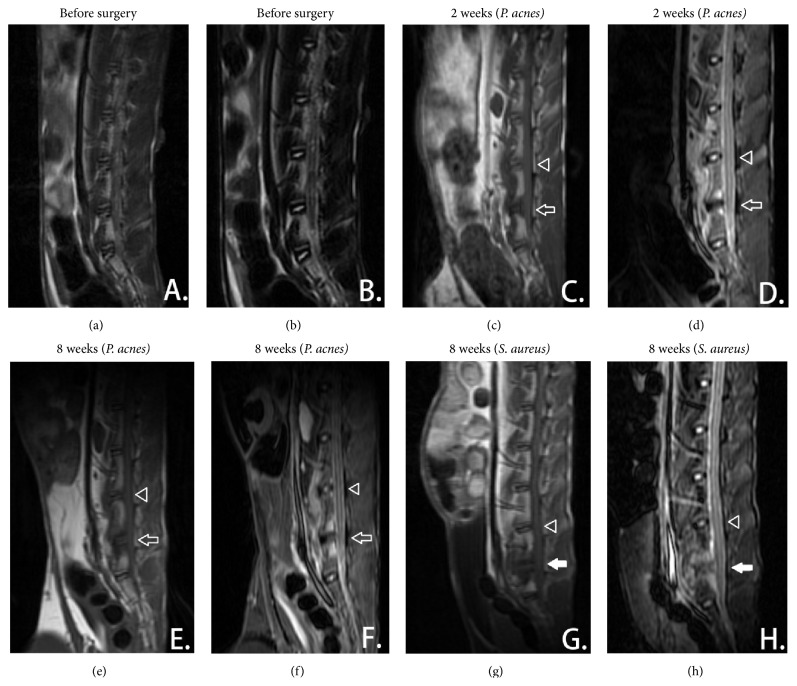
Signal changes were observed after the inoculation of the isolated wild-type strain of* P. acnes and S. aureus*. (a)~(b) Before the surgery, there was not any abnormality at T1WI and T2WI. (c)~(d) Since the second week, an obvious hypointense signal was observed on T1WI (c) and the hyperintense signal was found on T2WI (d) at the* P. acnes*-inoculated segment (L6~L7, indicated by a white arrow). Meantime, hyperintense signals of nucleus pulposus in T2WI disappeared, suggesting disc degeneration. (e)~(f) The signal changes remained constant until the eighth week at the end of follow-up. The volume of signal changes had increased from the second week to the eighth week. (g)~(h) The hyperintense signal changes were also observed in the* S. aureus*-inoculated segment (L6~L7, indicated by a white filled arrow) on T1WI (g) and T2WI (h); however, the definition of the endplates and vertebral body was less intact and more severe inflammatory signal changes were found. No significant signal changes were observed at the internal control segment of L5~L6 in all groups (indicated by a white triangle).

No abnormal signals were found before the surgery (Figures 2(a) and 2(b)). However, signal changes of cartilage endplates were detected at both the wild-type strain and standard strain of* P. acnes*-inoculated segment (L6~L7) since the second week, revealing hypointense signals at T1WI and hyperintense signals at T2WI (Figures 2(c) and 2(d)). Furthermore, the normal hyperintense signal of nucleus pulposus shifted into hypointense signal on T2WI, demonstrating a “black disc” which was considered as an indication of disc degeneration. All of these abnormal signals remained constant for eighth week until the euthanasia of the animals and the volume of signal changes had increased from the second week to the eighth week (Figures 2(e) and 2(f)). By contrast, the TSB-inoculated internal control segment was normal during the observation (Figures 2(c)–2(f)). According to Pfirrmann classification system, the severity of the intervertebral disc degeneration was V or IV grades in all of the wild-type strain or standard strain of* P. acnes-*inoculated animals.

In addition, similar signal changes were also found in the* S. aureus*-inoculated intervertebral discs, presenting as hyperintense signals at the endplates and hypointense signals at the nucleus pulposus on T2WI. However, the definition of the endplates and vertebral body was less intact and more severe inflammatory signal changes were found at the* S. aureus*-inoculated segment (Figures 2(g) and 2(h)).

### 3.2. Lower Death Rate in* P. acnes*-Inoculated Animals

All of the five* P. acnes*-inoculated animals remained alive until the end of follow-up. However, of the three* S. aureus*-inoculated rabbits, one died at the fourth week and another died at the fifth week (Table 1). Only one rabbit was still alive at the end of follow-up. Thus, the inoculation of* S. aureus* seemed more fatal than that caused by* P. acnes* (2/3* versus* 0/5, Table 1).

### 3.3. Disc Degeneration and Endplates Rupture at* P. acnes*-Inoculated Segment

**Figure 3:**
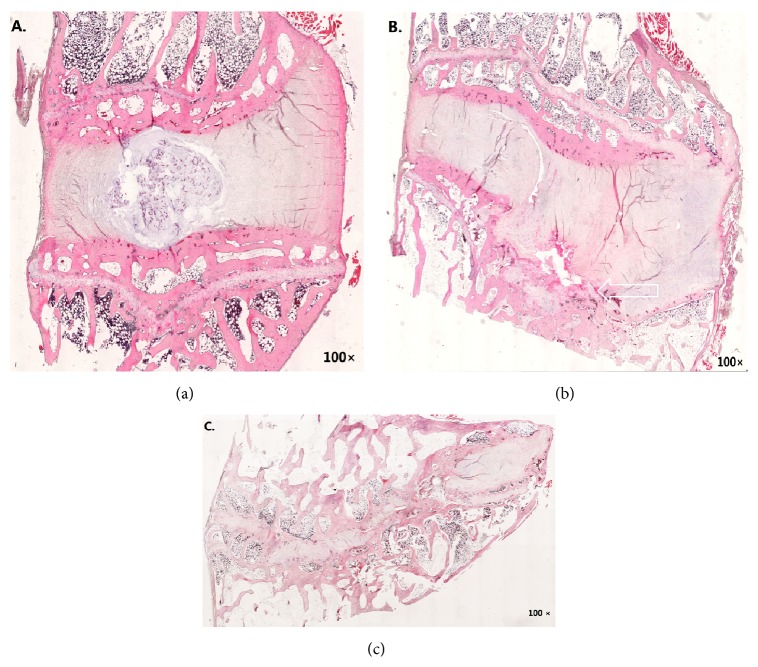
Eight weeks after the inoculation,* P. acnes*-inoculated intervertebral discs showed significant disc degeneration and endplates rupture but without severe discitis. (a) The intervertebral discs harvested from the TSB-inoculated internal control segment (L4~L5) had distinct nucleus pulposus and normal arranged annulus fibrosus. (b) The segment of wild-type strain of* P. acnes*-inoculated intervertebral discs (L6~L7) was demonstrated as disappearance of nucleus pulposus, endplates fracture (white arrow), disorganized annulus fibrosus, and partly cartilage proliferation. (c) By contrast, the intervertebral space significantly narrowed in* S. aureus*-infected intervertebral discs in coincidence with total cartilage tissue replacement and disappearance of nucleus pulposus and annulus fibrosus.

In histological examination, TSB-inoculated intervertebral discs showed normal anatomical structure, in which nucleus pulposus was enclosed with normal annulus fibrosus (Figure 3(a)). However,* P. acnes*-inoculated segment was depicted as endplates rupture and moderate disc degeneration with the disappearance of nucleus pulposus and disorganizing of annulus fibrosus (Figure 3(b)). Comparatively, intervertebral discs infected with* S. aureus* showed significantly narrowed intervertebral space and annulus fibrosus was completely replaced by cartilage, indicating severe pathological change by discitis (Figure 3(c)). Thus, it was reasonable to claim that* P. acnes* inside intervertebral discs would result in disc degeneration and endplates rupture rather than the severe discitis.

## 4. Discussion

The wild-type strain of* P. acnes* isolated from the patient with disc degeneration and Modic change resulted in the same signal changes when inoculated into the intervertebral discs of rabbits. Associated with histological examination, the signal changes were more reasonable to be considered as Modic change and disc degeneration. Furthermore, compared to the higher death rate and severe pathological change of discitis caused by* S. aureus*, the inoculation of* P. acnes* inside intervertebral discs did not result in severe discitis. Therefore, it was strongly indicated that* P. acnes* was one of the pathogens for Modic change and disc degeneration.

Modic change is the abnormal signals at the endplates and subchondral bone on MRI, which is always secondary to the disc degeneration [1]. However, it may be similar to discitis on MRI, especially the Modic-I change, and it is not easy to distinguish them in certain cases. Conventionally, typical pyogenic discitis will show hyperintense signals involving the intervertebral discs and vertebrae on T2WI and defect or erosion at endplates and vertebral body with/without the abscess surrounding the intervertebral discs [19]. Nevertheless, for most atypical discitis and discitis in early stage, hyperintense signals may only involve the endplates and subchondral bone but without any abnormality in disc and surrounding soft tissue [20]. Even in some reported* S. aureus*-infected rabbit models, no hyperintense signal changes were observed at intervertebral discs and surrounding soft tissue, which was also similar to this study's result [17]. Here, in this study, the outline of the endplates is more intact in* P. acnes*-inoculated segment and the inflammatory oedema seemed more severe in* S. aureus*-inoculated segment so that we are inclined to the Modic changes rather than discitis in* P. acnes*-inoculated segment.

Based on Koch's Postulates, we further believed that* P. acnes* was one of the pathogens causing Modic changes and disc degeneration. As one of the most important criteria to judge the relationship between a specific microbe organism and a specific disease, “finding abundant microorganism in organisms suffering from the disease” and “isolating and culturing this organism in pure culture medium” were the first and second criteria, and “cultured microorganism could cause disease when introduced into a healthy organism” and “re-isolated from the inoculated, diseased experimental host” were the third and fourth steps [21]. Previous study has demonstrated the existence of* P. acnes* in the nonpyogenic degenerated intervertebral discs [6, 8], and our previous study also proved a prevalence of 23.9% in degenerated discs [16]. Here, the MRI and histological evidence (Figures 2 and 3, resp.) further suggested that inoculated* P. acnes* inside animal's intervertebral discs would cause Modic change and disc degeneration. All of these data satisfied the first, second, and third criteria of Koch's Postulates and thus we could extrapolate that* P. acnes* has a strong connection with Modic change and disc degeneration.

Being different from* S. aureus*, which was well known as the most frequent pathogen in spondylodiscitis [22],* P. acnes* rarely caused pyogenic discitis. In a thorough review, only twenty-nine cases of* P. acnes*-induced spondylodiscitis were reported within the last 15 years [23]. On the contrary, the prevalence of* P. acnes* in nonpyogenic intervertebral discs was as high as 44% [6] and a similar prevalence was independently reported by other different research groups [7, 8]. One of the possible reasons may be the fact that* P. acnes* had different species and some of them are more virulent, but others may be less virulent [11], thus probably leading to different pathological change and dispersion of MRI image. Generally speaking, we favored that when the intervertebral discs are infected by* P. acnes* they would have more chance to result in the latent low-grade chronic inflammation [6], which is then represented as Modic changes and disc degeneration rather than the pyogenic discitis.

Recently, an illuminating animal paper tried to reveal the routine of how* P. acnes* gets access into the intervertebral discs [18]. Although the results did not support the hypothesis proposed by Albert et al. that* P. acnes* may intrude the intervertebral discs via circulation system [9], it proved that intervertebral discs are suitable for the growth and reproduction of* P. acnes*, because the reisolation percentage was as high as 61% after the inoculation of* P. acnes* directly into the intervertebral discs. More importantly, disc degeneration was also observed on the MRI after* P. acnes* inoculation, which was consistent with our results [18].

Unfortunately, it is still unclear how* P. acnes* induces the Modic changes. One viewpoint was that the propionic acid, the metabolite secreted by* P. acnes*, resulted in the dissolution of fatty bone marrow and bone, thus represented as Modic changes [10]. Another hypothesis was the local inflammatory activated by* P. acnes* [9].* P. acnes* has been proved to have a strong ability to stimulate monocytes to produce proinflammatory factors such as TNF-*α* and IL-1 *β* [24], so that these proinflammatory factors may be involved in the damage and oedema of endplates and subchondral bone marrow, which is displayed as Modic-I change on MRI [25]. Also, increased proinflammatory factors would lead to the disc degeneration [26].

During the isolation of* P. acnes* from human being, we followed previous method: muscle and ligaments were extracted as the contamination marker to avoid false-positive culture result [12, 16]. In addition, the reason that we used adjacent intervertebral disc for TSB inoculation is that it will keep the bacterium-inoculated and TSB-inoculated segments having the same surgical condition. Moreover, the purpose that sterilized TSB instead of saline was used as the solvent to prepare* P. acnes* suspension was to maintain bacterial viability. Although macromolecular substances in the TSB may have unpredictable effect, the TSB alone inoculation segment did not show any abnormal changes, suggesting that TSB used as the solvent was a practicable option. Finally, according to previous study, the penetration with 28-gauge needle had minor or no effect on disc degeneration in rabbits [27].

However, some limitations still exist in this experiment. First, other low-virulence anaerobic floras, like* coagulase-negative staphylococci*, were not tested in this experiment. In addition, observation duration was relatively short in this study. Long-term effect of* P. acnes* on intervertebral discs and endplates requires further study.

## 5. Conclusion

Based on Koch's Postulates,* P. acnes* has strong correlation with disc degeneration and Modic changes when inoculated into intervertebral discs, which is definitely different from discitis caused by* S. aureus* in histological examination and death rate. In clinical practice, one should be more aware of the presence of* P. acnes* in patients with Modic changes and disc degeneration.

## Figures and Tables

**Table 1:** Animal group information.

	Inoculated bacterial type	Numbers	Inoculated segment	Bacterial load	Dead or not
Group A	The wild-type strain of *P*. *acnes* isolated from patient	3	L6~L7	*P*. *acnes* suspension in sterilized TSB without bovine serum at 1 × 10^7^ CFU/mL with 25 *μ*L	All alive until the end of follow-up
			L5~L6	Sterilized TSB without bovine serum with 25 *μ*L	

Group B	The standard strain of *P*. *acnes* (ATCC 6919)	2	L6~L7	*P*. *acnes* suspension in sterilized TSB without bovine serum at 1 × 10^7^ CFU/mL with 25 *μ*L	All alive until the end of follow-up
			L5~L6	Sterilized TSB without bovine serum with 25 *μ*L	

Group C	The standard strain of *S*. *aureus* (ATCC 25923)	3	L6~L7	*S*. *aureus* suspension in sterilized saline at 1 × 10^7^ CFU/mL with 25 *μ*L	One died at the fourth week One died at the fifth week One alive until the end of follow-up
			L5~L6	Sterilized saline with 25 *μ*L	
